# The Society of Homœopathicians

**Published:** 1895-01

**Authors:** B. Fincke

**Affiliations:** Brooklyn, N. Y.


### THE SOCIETY OF HOMŒOPATHICIANS.—A PROTEST.

When I accepted the honor of being duly elected a charter member of the Society of Homœopathicians and the Declaration of Principles, Rules, Constitution, and By-Laws, which I considered as the best that had ever been drafted, I had no idea that their publication would be preceded by a preamble which from first to last I must emphatically disavow and disapprove. I thought a *modus vivendi* could be found to continue the membership in either body, the I. H. A. and the S. H., because both advocate the same object, viz., the study and dissemination of the principles of Homœopathy as promulgated by Samuel Hahnemann, but this is made impossible after the falsehoods contained in that preamble. Lest I should be mistaken for a party to it, I hereby distinctly avow my continued allegiance to the International Hahnemannian Association, and declare that I consider myself free from any further connection with the Society of Homœopathicians, if that odious preamble is not retracted publicly in the same journal where it was published.

B. Fincke.

Brooklyn, N. Y., Nov. 26th, 1894.

